# A Case of Acne

**Published:** 1893-02

**Authors:** D. C. McClaren

**Affiliations:** Ottowa, Can.


### A CASE OF ACNE.

D. C. McLaren, M. D., Ottawa, Can.

On December 26th, 1889, Miss H., aged twenty-seven, came to me for treatment for the worst facial acne I have ever seen. There were thick clusters on both cheeks, across the nose, on the chin and forehead. They began on the forehead originally as simple red acne some eight years before, and for five years had been very disfiguring. It grew so much worse from a recent cold that she decided to seek treatment for the disorder. At this time the eruption was pustular, with stinging pains when near the stove. She was always better in summer, though the face felt better in the cool air of winter; the face was greasy in summer; hair dry and turning slightly gray; fine, dry dandruff. Any exercise flushes the face and causes stinging pain; also, stooping over makes the face hot and red. Washing in tepid water gave relief, but in either hot or cold water aggravated. Mental condition irritable.

Began the treatment with one dose of Belladonna$^{40m}$, which greatly improved the acute symptoms. The next remedy was Sulphur, given on January 6th, 1890. After this the eruption became much thicker, but with relief of an old tired feeling which had not been mentioned at first. After about six weeks there was considerable disturbance of the general health, for which she received a dose of Rhus, which was evidently a mistake, as three weeks later the Sulphur was repeated (March 13th), with benefit. On May 19th, however, I gave Rhus again; after this the menses, which had been greatly disturbed during the previous five months, became more settled, but with a new characteristic, viz.: flowing only in daytime. With this as a key-note, she got Causticum on July 5th. There was no medicine given until September 29th, when she again received Rhus for the acne, which now began to recur less frequently, became much smaller, and not so pustular. The face became hot and red from a cold, for which Belladonna was given October 27th, followed by Calcarea$^{85m}$ on November 17th, which improved the case wonderfully. From then till May 7th, 1891, she got Belladonna and Calcarea at intervals of about six weeks, and during all this time there was steady improvement, though slow. On July 28th, gave a dose of Psorinum$^{50m}$, which was allowed to act undisturbed for a little over four months. Then a dose of Sulphur on December 3d, 1891, virtually ended the case, and the patient is now practically cured, though it will take a long time yet for the skin of her face to get over the scars caused by such a long-continued eruption.

---

Dr. Farley—The writer complained of that result as a slow one. It does not seem so to me. The result was rapid for such a case.

Adjourned to 8 P. M.

